# Crohn’s Disease: Potential Drugs for Modulation of Autophagy

**DOI:** 10.3390/medicina55060224

**Published:** 2019-05-29

**Authors:** Nursyuhada Azzman

**Affiliations:** Faculty of Pharmacy, Universiti Teknologi MARA, Cawangan Pulau Pinang Kampus Bertam, 13200 Kepala Batas, Pulau Pinang, Malaysia; nursyu437@ppinang.uitm.edu.my

**Keywords:** autophagy, signalling pathways, Crohn’s disease, drugs, adherent-invasive *E. coli*, inflammatory bowel disease, cytokines, ulcerative colitis

## Abstract

Autophagy is an intracellular process whereby cytoplasmic constituents are degraded within lysosomes. Autophagy functions to eliminate unwanted or damaged materials such as proteins and organelles as their accumulation would be harmful to the cellular system. Autophagy also acts as a defense mechanism against invading pathogens and plays an important role in innate and adaptive immunity. In physiological processes, autophagy is involved in the regulation of tissue development, differentiation and remodeling, which are essential for maintaining cellular homeostasis. Recent studies have demonstrated that autophagy is linked to various diseases and involved in pathophysiological roles, such as adaptation during starvation, anti-aging, antigen presentation, tumor suppression and cell death. The modulation of autophagy has shown greatest promise in Crohn’s disease as most of autophagy drugs involved in these diseases are currently under clinical trials and some has been approved by Food and Drug Administration. This review article discusses autophagy and potential drugs that are currently available for its modulation in Crohn’s disease.

## 1. Introduction

Crohn’s Disease (CD) is one type of chronic relapsing inflammatory bowel disease (IBD) which involves the inflammation of any part of the digestive system between the mouth and anus. It occurs most commonly at the colon, ileum or both [1]. The annual incidence of CD was found to be ranging from 2–20 cases per 100,000, and a significant increase in the incidence of IBD was reported from 75% of CD studies with no decreasing trend since 1980 [2].

The disease is thought to be higher in West countries such as Europe (322 per 100,000 in Germany) and North America (319 per 100,000 in Canada) [3]. However, recent studies showed that Europe had a decreasing trend of IBD incidence while an increasing trend is showed by Asia for the last two decades [4,5]. There is an increasing trend of CD in Asia compare to ulcerative colitis (UC) moreover in developed countries such as Hong Kong, Korea and Japan [6].

At present, there is no cure for CD and the goal of current treatment is to induce and maintain steroid free remission period while avoiding complications from the treatment or the disease itself. A critical observation regarding current drug treatment is needed as more than half of UC patients will usually have a relapse in the same year after flare [7].

There is a need of research to optimize medical therapies as highlighted by The Crohn’s and Colitis Foundation of America [8] as the process and cost of making new drug is lengthy, expensive and often associated with high failure rates. To improve the efficacy of the current medications, knowledge on their mechanism of actions need to be obtained. Therefore, this review gives an insight of IBD drugs that have been linked to modulation of autophagy, a cellular process that is related to CD pathogenesis together and their current mechanism of action.

## 2. Autophagy

Autophagy is a process of self-digestion during which cytoplasmic constituents are degraded within lysosomes. There are three main type of autophagy which are classified according to the different pathways by which cargo is delivered to the lysosome or vacuole. They are chaperone-mediated autophagy (CMA), microautophagy and macroautophagy [9].

CMA is involved in degradation of single, soluble proteins containing the KFERQ pentapeptide sequence and is only described in mammals. Micro- and macroautophagy on the other hand occur in a wide range of eukaryotes including plant, mammals and fungi. They lead to the degradation of certain portions of the cytoplasm including cell organelles [10]. Microautophagy occurs by pinching off the lysosomal membrane, allowing the sequestration and elimination of cytoplasmic components. Unlike CMA and microautophagy, only macroautophagy generates a new organelle known as an autophagosome. This structure delivers a large number of different cargo molecules into the lysosome [11,12].

Macroautophagy is the best characterized process and most widely studied among the three main forms of autophagy, therefore most of studies referred macroautophagy as autophagy [13]. Initially, this process was described in cells under stress condition such as starvation or accumulation of toxic components including proteins or damaged organelles. By degrading the cellular substrates, the fatty acids and amino acids being produced will be used to synthesize new proteins or oxidized by mitochondria to produce adenosine triphosphate (ATP), providing the energy to the starved cells for survival [13,14].

Recent studies showed that autophagy is also playing an important role in several physiological processes such as normal development, differentiation and tissue remodeling, which support the expansion of life span as well as the maintenance of cellular homeostasis and tumour suppression. Autophagy is also involved in the innate immune response by removing invading pathogens [12].

### 2.1. The Process of Autophagy

**Figure 1:**
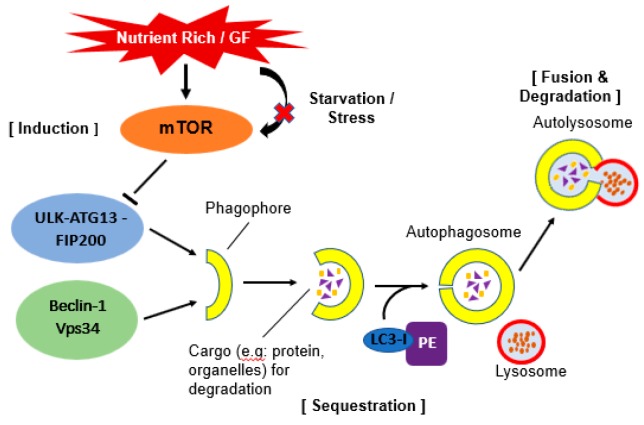
Schematic illustration of autophagy. Under stress or starvation, mTOR will be inactivated hence initiate the activation of autophagy by phosphorylation of ULK-ATG13-FIP200 complex. When this complex interacts together with Beclin-1-Vps34 complex, it will form a double layer membrane known as phagophore. Next, Atg4 will cause cleavage of the microtubule-associated protein 1 light chain 3 (Atg8/LC3) and formation of cytosolic LC3-I protein. This complex then conjugated to phosphatidylethanolamine (PE) to form membrane bound LC3-II. The conjugation will help LC3 to determine its site of lipidation. As the membrane grows, it will enwrap certain portion of the cytosol and formed autophagosome which contains autophagic cargo. When lysosome fuse with this autophagosome, it will release hydrolase and cause degradation of the vesicle content. A process known as autolysosome.

Autophagy requires several autophagy-related gene (ATG) proteins. These ATG proteins function in several steps of the autophagy process. There are a few sequential steps which occurs in autophagy. They are induction, sequestration, fusion, formation of autolysosome and degradation of contents (Figure 1).

#### 2.1.1. Induction of Autophagy

Autophagy can be induced by various stress conditions. A key regulator of this process is mammalian target of rapamycin (mTOR). Under nutrient-rich conditions, mTOR promotes the transcriptional activity of Foxk proteins which act as repressors of autophagy. Through this mechanism, mTOR limit the basal levels of autophagy by repressing essential autophagy genes [15]. Under starvation conditions, mTOR will be inactivated and its repression of autophagy is relieved [16].

The inactivation of mTOR leads to dephosphorylation events which then trigger transcriptional activation of autophagy genes that are involved in macroautophagy machinery [17]. During starvation, inhibition of mTOR causing activation of Atg1 homologs known as Unc-51- like kinase 1 (ULK1) and -2 (ULK2). Both Atg1 homologs will then phosphorylate Atg13 and FIP200 which are essential for autophagy activity. These complexes will then localize to the phagophore [18]. The depletion of essential nutrients triggers induction of autophagy. In yeast and plants, this is believed to occur due to lack of nitrogen or carbon [19].

In mammals, it is quite complicated as depletion of total amino acids that release zinc from degradation protein causing upregulation of autophagy, however this depends on cell type as amino acid metabolism differs greatly among tissues [20]. Furthermore, the protein containing ULKs-Atg13-FIP200 seems to be stable regardless of nutritional conditions in mammalian cells [18]. Recently, there has been evidence suggesting that autophagy induction with amino acid signaling pathway involves Beclin 1 and class III phosphatidylinositol 3 (PI3)-kinase [21].

#### 2.1.2. Sequestration and Autophagosome Formation

A double membrane vesicle called an autophagosome forms in the cytosol and sequesters proteins and organelles. The origin of this autophagosome is uncertain however, it is postulated that it may arise from endoplasmic reticulum, mitochondria and plasma membrane [11]. The formation of the autophagosome is coordinated by Atg proteins in complex form. Particularly, Atg 5 and Atg12 will be conjugated and cause Atg8 homolog (LC3) recruitment. Atg8 is a ubiquitin-like protein usually conjugated with phosphatidylethanolamine (PE) and causes the elongation of phagophore or isolation of the membrane at the autophagic membrane [22]. Atg14 forms a complex with Beclin-1. Beclin-1 is released from Beclin-2 to activate autophagy during starvation [23].

#### 2.1.3. Docking and Fusion with Lysosome (Autolysosome)

Upon completion of the autophagosome, Atg4 will cleaves Atg8 from PE and Atg8 is released back to the cytosol [24]. Autophagosome will then fuse with the lysosome and this process requires the small GTPase Rab7 and lysosomal membrane protein (LAMP-2) in mammalian cells [25]. It is postulated that autophagosome will fuse first with endosomes in order to provide the required lysosome fusion machinery and become amphisome. After that, fusion with the lysosome can occur. The autolysosome will degrade all the inner membrane and cytoplasm-derived material by lysosomal/vacuolar hydrolases [26].

## 3. Autophagy and CD

**Figure 2:**
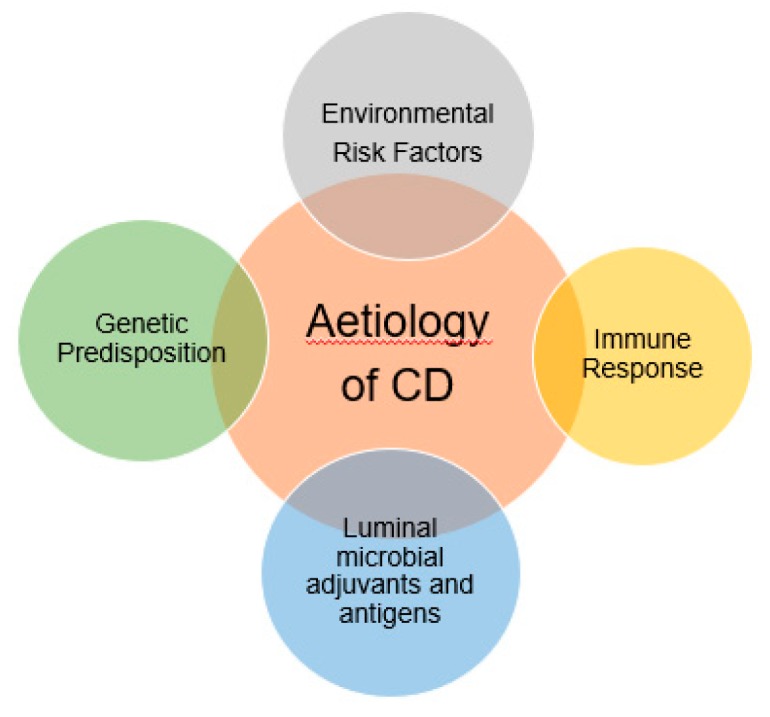
Multifactorial risk which are related to the aetiology of Crohn’s disease. A genetically susceptible host usually more prone to get excess inflammation of the bowel tracts due to the aggressive immune response.

Crohn’s Disease (CD) is a chronic inflammatory bowel disease characterized by inflammation, neutrophil influx in the intestinal epithelia and ulceration. The pathophysiology of the disease is unclear however it may involve excess inflammatory responses, impaired clearance of intracellular bacterial and abnormal Paneth cell granule secretion [27]. The most widely accepted theory regarding pathogenesis is due to the overly aggressive acquired immune response most specifically T cell due to the enteric bacteria in genetically susceptible hosts (Figure 2) [28].

Previously, several studies have shown that imbalances of gut microbiota ecosystem may lead to inflammation in response to autoimmunity in genetically susceptible host [29]. However, in normal condition, the gut microbiota which consists of two type of phyla known as *Firmicutes* and *Bacteroidetes* are non-pathogenic. A majority of them help to aid the metabolism of nutrients and drugs. Furthermore, they prevent colonization and invasion of pathogenic microorganisms by controlling the overgrowth of pathogenic strains by inducing immunoglobulin. In addition to these, gut microbiota is also involved in the alteration of immune response which are related to innate and adaptive immune systems [30].

In the intestinal mucosal, activation of dendritic cells (DCs) by *Bacteriodes* induce plasma cells to express secretory IgA (sIgA) which in turn coat the gut microbiota from degradation by bacterial proteases. This subclass known as sIgA2 is different from sIgA1 phenotype where sIgA1 may move to the circulation while sIgA2 remains in the intestinal lumen. A proliferation-inducing ligand (APRIL) produced by intestinal epithelial cells (IECs) will restrict the translocation of gut microbiota from the intestinal lumen to the circulation by a class switch mechanism that will maintain the sIgA1 subtype [31].

The production of IgA also is believed occurred due to activation of My-D88 signalling by gut microbiota in follicular and lamina propria regions of DCs. The activation of DCs by gut microbiota also occurred in Peyer’s patches where CXCL13, TGF-β, and B-cell activating protein (BAFF) are expressed and increase production of IgA [32]. The interaction of sIgA with DCs induces inhibitory signals that eventually reduce excessive immune response. sIgA also prevents the attachment of pathogen to intestinal epithelial cells by acting as a competitive inhibitor to the site of binding on the epithelial cells due to the structure of its oligosaccharide side chain which shares a high degree of similarity with the luminal face of the intestinal epithelium of the host cells [33].

This will eventually prevent the attachment of pathogen or toxins making sIgA a component of innate immune system. sIgA is found abundantly in mucosal secretion and is believed to be working to limit the access of allergen to lamina propria by inhibiting the activation of mast cells [34]. In short, sIgA demonstrates varieties roles to maintain the mucosal homeostasis as it downregulates pro-inflammatory responses in presence of pathogenic bacteria, prevent the attachment, limit the allergens, and at the same time influences the intestinal microbiota constitution.

Recent genome-wide associate scanning (GWAS) include several genetic-relationship with CD susceptibility due to single nucleotide polymorphism in genes involved in the innate immune response (*NOD2*), specifically, acquired T cell response (*IL23R*) and autophagy (*ATG16L1, IRGM*) [35]. The most notable microorganism which contributes to CD is *E. coli* strains with an adherent and invasive phenotype (AIEC) [36]. The invasion of this bacteria is related to the CD-associated gene variants as a recent study relates enhanced replication and survival of AIEC strain LF82 with *ATG16L1* and *IRGM* deficient cells. Both of *ATG16L1* and *IRGM* are autophagy genes [37].

Nucleotide oligomerisation domain protein 2 (NOD2), a member of NLR (NOD-like receptor) is an intracellular pathogen molecular sensor that plays important roles in innate immune response as it recognizes muramyl dipeptide (MDP), a component of the peptidoglycan present in the bacterial cell wall [38]. Studies have shown that NOD2 is important in the regulation of microbiome, bacterial autophagy, viral recognition and can act as therapeutic target for CD [39,40].

A recent study by Stevens et al. [41] demonstrated that there is binding between leucine rich repeat (LRR) domain within NOD2 with Vimentin, an intermediate filament protein. The majority of NOD2 binds to the cytoskeleton and inhibition of Vimentin by Withaferin A causes relocalisation of NOD2 to the cytosol. The inability of Vimentin to interact with NOD2 contributes to mislocalisation of L1007fs and R702W NOD2 variants. This leads to disruption of NOD2 activities such as NF-κB activation, autophagy induction and bacterial handling as these activities are dependent on NOD2 plasma membrane localisation.

NOD2 stimulation with MDP also is associated with induction of autophagy in human, monocyte-derived dendritic cells (DCs) and affects bacterial handling and antigen presentation. The process requires NOD2 signalling mediator RIPK-2 in addition to PI3K and the autophagy proteins such as Atg5, Atg7 and ATG16L1 and independent from Toll-like receptor (TLR) signalling [42].

ATG16L1 is one of the Atg proteins that form a complex with Atg12 and Atg8 which is involved during autophagosome formation and LC3-lipidation. The complex localises at the outer surface of the isolation membrane and upon completion of autophagosome, it dissociates. The correct localisation of ATG16L1 during this LC3-lipidation is vital for the appropriate autophagosome formation, and any overexpression of it will reduce autophagy [43]. There is also evidence related to CD shows in ATG16L1-deficient macrophages, high amounts of inflammatory cytokines interleukin 1-beta is being produced after stimulation with the toll-like receptor 4 (TLR4) ligand lipopolysaccharide (LPS) [44].

One of the genes that activate autophagy as a mechanism for the elimination of invasive microorganisms is immunity-related GTPase family M (IRGM). This gene belongs to the p47 immunity-related guanosine triphosphate family [45]. Depletion of IRGM in macrophages and human intestinal cells resulted in defective autophagy and leads to increase of intracellular AIEC (Lapaquette et al., 2010, Lapaquette et al., 2012) [37,46]. This result shows that the impairment of autophagy genes allows AIEC to replicate and survive, leading to the progression of CD.

Although there is genetic predisposition linked with CD, the presence of the gene for instance NOD2 is not necessarily sufficient to cause CD. Many people with CD do not have this gene and most people who have NOD2 mutations do not develop the disease. It is thought that there must be other genetic abnormalities or maybe environmental factors that set pathogenesis of IBD [47]. Polymorphism in genes might be the potential factor as discussed earlier and it is related to autophagy genes, *ATG16L1* and *IRGM* which are involved in the pathogenesis by affecting the host cell responses to intracellular microbes [48,49].

## 4. CD and Adherent/Invasive *Escherichia coli* (AIEC)

As discussed earlier, the pathogenesis of CD is not really understood but the strongest correlation between genetically susceptible individuals whom in an environmental and/or infection from commensal intestinal bacteria which involved autoimmunity response that triggering intestinal inflammation and cause tissue damage [50,51]. It has been postulated that several of these infectious bacteria which are known are including *Listeria monocytogenes, Mycobacteria paratuberculosis, Klebsiella pneumonia* and various strains of adherent/invasive *Escherichia coli* (AIEC), however, the evidence to support each of these infectious agents is inconclusive [52].

Recently, more studies have been conducted that found strong evidence between the pathogenesis of CD with mucosa-associated AIEC strains. For instance, few independent studies done in Europe and United States have identified the presence of intramucosal *Escherichia coli* or mucosa-associated *E. coli* with invasive properties in CD patients [53,54]. This is also supported by recent data which showed that AIEC is able to target M cells on human Peyer’s patches via the expression of long polar fimbriae that eventually allowing them to translocate across the intestinal epithelial [55]. The adhesion of AIEC is also dependent on the abnormal expression of carcinoembryonic antigen-related cell adhesion molecule 6 (CEACAM6) by illeal epithelial cells in CD patients and acts as receptor which recognized type 1 pili variant expressed by AIEC for the adhesion process [56].

Other than this, the mechanism on AIEC invasion also involved macrophages which help them to survive extensively and induce high secretion of TNF-α as its correlated to the intracellular replication of AIEC [57,58]. Moreover, the replication of AIEC within these regions such as in epithelial and dendritic cells is being assisted by the defects of autophagy due to mutation in ATG16L1, IRGM and NOD2 7) [37,42]. Macrophages in CD patients have been associated with one of AIEC phenotype strain known as LF82 strain.

This type of AIEC is able to penetrate enterocytes, survive and then replicate within macrophages without causing host cell death [59]. AIEC LF82 also expressed type 1 pili and several outer membrane proteins (OMPs) for adhesion during the invasion [60] making it a perfect candidate to be recognized by CEACAM6 [61]. AIEC-infected macrophages do not undergo apoptosis and accumulate to form granulomas [59]. AIEC LF82 is believed to induce this and cause the aggregation of the infected macrophages to form multinucleated giant cells together with the recruitment of lymphocytes [62].

Although impaired macrophage TNF-a secretion has been reported in *Legionella pneumophila, Coxiella burnetii* and *Brucella species* as the secretion of TNF-a by immune cells provides a suppressive environment which inhibit their replication [57]. This is however not applicable in the case of *Mycobacterium* and AIEC LF82 where in contrast to this, another study done demonstrate that TNF-*α* is showing an opposite effect on intracellular replication such as *Mycobacterium* where exogenous TNF-*α* enhances and accelerate the multiplication *Mycobacterium* [63].

To support this, another study showed that the replication is being inhibited upon introduction of anti-TNF-*α* monoclonal antibodies in macrophages infected with *M. tuberculosis* [64]. Like *Mycobacterium*, TNF-*α* also promotes AIEC LF82 intramacrophagic replication within macrophages and may harm the mucosa of CD patients. This is believed to occur due to the activation of antiapoptotic signals by TNF-*α* [65] being secreted from the AIEC-infected macrophages where it interferes with apoptosis, thus preventing cell death to support the intramacrophagic replication niche for AIEC itself [57].

A study done by Donohoe et al. [66] demonstrated that some microbiome generate butyrate in the colonocyte function to maintains homeostasis and acts as energy source which prevents autophagy rather than as HDAC inhibitor. This is supported by several studies which showed that there are high densities of microbes in the lumen of the colon that are involved in the conversion of fermented dietary fibre into butyrate at very high concentrations. Specifically, these bacteria belong to genera such as *Eubacterium*, *Butyrivibio* and *Clostridium* [67].

Short-chain fatty acids (SCFAs) is the end product of fermentation from dietary carbohydrates. Among SCFAs such as acetate, propionate and butyrate help to maintain normal large bowel function and in particular, butyrate has been implicated to prevent pathologic conditions [68] by giving protection against colitis and colorectal cancer as it is the preferred energy source for colonocytes [69].

Furthermore, the concentrations of SCFAs is depending on the microflora activity together with nutrient condition which can be affected by dietary changes [70]. At first it was thought that an increase of butyrate concentration is beneficial as it reduces bowel inflammation and prevent development of colon cancer [69]. Therefore, a strategy to enhance its production by probiotics and prebiotics through diet might be an ideal approach to prevent colonization of intestinal pathogens [70].

Subsequently, it was found that there is correlation between one type of *E. Coli* known as Enterohaemorrhagic *Escherichia coli* (EHEC) with the formation of butyrate by the lumen microflora. A study by Jubelin et al. [71] found that although the growth of EHEC is being inhibited at a high concentration of SCFAs, the presence of SCFAs enhanced the expression of its virulence genes which are required for induction of attaching and effacing (AE) lesions together with cell adhesion. Particularly, the expression of these virulence genes is enhanced by butyrate even at low concentration.

The levels of SCFAs were found to be higher in proximal colon, and less in other region [72]. It was then found that the expression of the virulence genes known as LEE (Locus of Enterocyte Effacement) in EHEC occurred at low level of SCFAs between 6.25–25 mM without affecting bacterial growth. Concurrently, the growth was only being inhibited at 50 mM or higher [73].

Interestingly, although it is known that there is involvement of entire gastrointestinal (GI) tract in CD pathogenesis, it is however most common in the ileocecal area where there is presence of AIEC [53] which gives idea that there might be possible correlation between concentration of butyrate with expression of AIEC within the area. Nakanishi et al. proposed that butyrate acts as a signalling molecule which help to trigger the expression of adherence-related genes and initiate colonization of the pathogens in the distal ileum [73].

The response of EHEC towards butyrate is defendant to Lrp, a leucine-responsive regulatory protein which is the central for AE lesion formation and regulates the expression of LEE gene known as *Ler*. Interestingly, *Ler* not only regulates the expression of the elements in the AE phenotype but also in other LEE-encoded factors which are not involved in AE lesion formation [74].

## 5. Current IBD Drugs in CD

The current treatment of CD is according to the severity of the disease, which is necessary to design an appropriate treatment plan. Two types of assessment scores which are widely being used are Clinical Trial Crohn’s Disease Activity Index (CDAI) and the Harvey-Bradshaw Index. However, more convenient methods are usually preferred in clinical practice [75].

As the etiology of CD is quite complex and involved multi-factorial risks such as gene, environment and autoimmunity (Figure 2) therefore there is potential for new therapeutic targets [76]. In general, the disease severity, behaviour and location determined the treatment plan while not forgetting any previous treatment failure to plan a future treatment strategy [77]. Therefore, the most relevant treatment plan is to individualize treatment which can be done according to the clinical response and tolerance of a patient.

There are currently two strategies in CD treatment, but the most current principle is the “step-up” strategy, which is using safer, less expensive drugs with less adverse effects than more potent but potentially more toxic drugs in order to induce and maintain remission. Only patients who had been identified to get benefit from the conventional treatments will be chosen. Another strategy is known as the “top-down” strategy which involves suggesting the use of more potent treatment options early in the treatment, especially to those with severely active CD and an increased risk of complication. The efficacy of both strategies however is not convincing enough to draw a conclusion as it is not appropriate for all patients [77].

Despite the chosen strategy, patients receiving treatment should be evaluated one or two weeks after the treatment started and followed up regularly. The intervals of follow-up are usually individualized depending on the chosen treatment and patient characteristics. It is anticipated that improvement can be seen in the first 2–4 weeks and the maximal effect is gained within 12–16 weeks of therapy. The remission induction therapy is followed by maintenance therapy only after clinical remission is achieved. In any treatment failure, it is necessary to consider alternative strategies [75].

For the time being, medication being used for treatment in CD are only involving the use of anti-inflammatory agents usually corticosteroids group and immunosuppressant. All of the treatments aim to stop the inflammatory process, relieving the symptoms by inducing and maintaining the remission stage and to avoid surgery wherever possible. Some of the antibiotics may act as immunomodulators other than giving their antimicrobial effects [78]. Other than that, there is also infliximab, a monoclonal antibody drug which target against TNF-α which can be used in patients who are not responded to aminosalicylates, antibiotics, corticosteroids, or immunomodulators [79].

Recently, there has been an update as anti-TNF alpha agents such as Adalimumab, Infliximab and certolizumab pegol have been approved to treat CD in the case of corticosteroids resistance. These agents could also be used during refractory to Thiopurines or Methotrexate. However, the usage of certolizumab pegol in CD approved only by FDA [80]. For patients with active CD either during the induction of symptomatic response or remission period, natalizumab, an anti-integrin can be used as it is more effective than placebo. In the cases where previous treatment such as the use of corticosteroids, methotrexate, thiopurines, or anti-TNF inhibitors failed, patients with moderate-to-severe CD can use ustekinumab, an anti-IL-23 as an option [81].

## 6. Modulation of Autophagy in Current CD Drugs

As previously discussed, the induction of autophagy in CD as a rational therapeutic strategy has attracted interest in recent years. Rapamycin (Sirolimus) and its analogue such as Temsirolimus is showing a potential benefit to CD patients as it improves the disease symptoms [82]. Moreover, it was shown that Rapamycin also seems to be an effective rescue therapy in children with severe IBD refractory by inducing clinical remission and mucosal healing [83].

Contradictory to these studies, Everolimus, the analogue of Rapamycin was shown to be less efficacious compare to Azathioprine and placebo even the safety and tolerability were similar [84]. Despite its potential as an autophagy inducer, a study by Reinisch et al. [84] failed to show the effect of Everolimus on the mTOR pathway in an experimental model for CD. The use of Rapamycin is still in clinical trials and the safety profile together with the mechanism of action for CD is not yet established.

Therefore, further studies with current medications which have been approved by FDA to be effective and safe in CD might be more favourable. There might be a potential that they might modulate autophagy as one of their mechanisms of action. A study by Nys et al. [85] highlighted the emerging role of autophagy and the potential therapeutic value of several drugs used in the treatment of CD either they induce autophagy as part of their mechanisms or as side effect of the drugs. Moreover, Nys and colleagues also showed that autophagy may act as a regulator of immune/inflammatory responses together as a stress-responsive mechanism (Table 1).

Following anti-TNF-α, the emergence of new classes of medication such as anti-IL-23 and anti-Integrin has shown great potentials in treating CD. Moreover, these two classes recently had been approved FDA and studies have shown that they are safe and effective to treat patient that is resistant to other medications with lesser side effects [81] It is believed that IL-23 and TNF-α play a vital role in driving inflammation and is correlated as IL-23 could abrogate the apoptosis induce by anti-TNF-α [91]. Few anti-integrins has been approved for CD where the first one is Natalizumab followed by Vedolizumab. However, the use of them is limited due to adverse effects [92].

### 6.1. Antibiotics, Thiopurines and Corticosteroids in CD

The use of antibiotics in CD has been a debate as there is no solid evidence that antibiotics therapy improves the course of CD, except for some evidence regarding Metronidazole which showed improvement, or its analogue could improve or reduce the probability of disease recurrence after surgical resection in ileal or ileocolonic CD. Although some clinical trials have been conducted regarding perianal CD with the use of Metronidazole for the past 10–15 years and Ciprofloxacin has been added as second antibiotic which proven to be effective, however, the reason behind this previously was unknown [47].

Recently, a study conducted by Tsuboi et al., discovered that the combination of Metronidazole and Ciprofloxacin reduces the expression of *Ang4*, *Retnlb*, *Itln1* and *Itln2* by the gut microflora and bacteria. Furthermore, knock-out mice of *Atg7* gene showed less efficient mucous that exacerbated colitis by the invasion of microbiota. The number of several bacterial species decreased following the treatment of these antibiotics. Interestingly, the level remains higher in the mice with *Atg7* knock-out compared to the mice without the genetic defect [89]. 

The use of Clarithromycin is more favourable in leukaemia cancer cells as the modulation is different from the other antibiotics. It is believed that inhibition of autophagy will lead to the apoptosis of myeloid leukaemia cells [90]. The induction of autophagy by thiopurines is also being recognized as there is involvement of the drug as an immunosuppressant, however, the mechanism remains unclear [85]. The autophagy is believed to become a compensatory response that protects the liver from side effects of Thiopurines such as myelosuppression and hepatotoxicity [93].

Wang et al found that glucocorticoids inhibit antimicrobial autophagy in macrophages after mycobacterial infection by decreasing the number of LC3-II formation that attached to the membrane of autophagosomes. The inhibition has been observed both in Dexamethasone and Hydrocortisone. The impairment of mycobacterial phagosome and autophagy in macrophages resulted in the survival of mycobacterial. Combination of glucocorticoids with other treatments may increase risk of infection in Tuberculosis [88].

The use of Corticosteroids has been proven to be effective for CD and UC patients. However, the treatment works only at initial phase where subsequently patients developed dependence towards Corticosteroids and required frequent operation which indicate poor prognosis towards the treatment [94]. Although the mechanism of it in CD is unknown, it is believed that it triggers cell death by induction of autophagy causing excessive death in the ileal and colonic epithelium [95].

### 6.2. Anti-TNF α, AIEC and Autophagy in CD

As previously discussed, there is inappropriate increase of immunogenic response against commensal bacteria and pathogen such as AIEC in the intestine that cause inflammation. It could be due to exaggerated inflammatory responses such as excessive production of TNF-α induced by AIEC and failure of autophagy to eliminate this pathogen worsening the condition which leads to manifestation of CD. Although currently there is no study in CD that shows linking between anti-TNF α with autophagy, study by Saito et al. [96] has shown that presence of TNF-α in autophagy-deficient intestinal epithelial cells cause them to lose their adhesive capacity. Therefore, the defect helps AIEC to invade and replicate, which eventually releasing more TNF-α.

Furthermore, because of autophagy defect occurs in macrophages of AIEC, the role of TNF-α in this condition is mainly to trigger anti-apoptotic signals to help AIEC replication and does not involve suppression of autophagy. The treatment of anti-TNF-α can be considered to inhibit the invasion and replication of AIEC itself. Moreover, the idea of combining anti-TNF α with autophagy inducer such as Rapamycin or any current drugs for CD which induce autophagy might be beneficial in the normal epithelial cells without any defects of autophagy.

As genetics play a major role in controlling our physiological response, the modulation of genes might be one of the key factors in treating CD. Furthermore, there are few genes controlling autophagy such as Bcl-2 family (e.g., Beclin-1, Bcl-2, Bcl-xL) and the defect of genes cause expression of AIEC (e.g., NOD2, ATG16L1, IRGM) which therefore give implication that their mutations is one of the reasons that exacerbate CD.

Given this idea, it was found that one promising drug which is currently being used for CD, Mesalamine (5-ASA) derivative known as ATB-429 might cause both of our therapeutic aims which is to induce autophagy and reducing TNF-α. Interestingly, the mechanism of action by this drug occurs at colon epithelial cells as it lowers proliferation and induces protective autophagy by releasing hydrogen sulphide (H_2_S) via AMPK and inhibition of mTOR [97]. Moreover, ATB-429 also reduces the expression of TNF-α [86].

### 6.3. Mesalamine and Autophagy in CD

Mesalamine (Mesalazine, 5-ASA) is a bowel specific aminosalicylate drug which has local action in the gut and is therefore claimed to have less systemic side effects but it the mechanism of action is not clear. Like corticosteroids, the use of mesalamine as an anti-inflammatory in CD remains controversial and a study found out that mesalamine is not effective to induce remission in active CD [87].

A recent update by Williams et al. [97] however examined the role of Mesalamine in CD and found data that support the use of it following surgically induced remission of CD together with its potential as prophylaxis for colorectal cancer and dysplasia. Furthermore, studies have shown that mesalamine acts at molecular level by affecting the production and action of several type of key-inflammatory cytokines such as interferon-gamma (IFN-γ), interleukin 1-beta (IL-1β) and interleukin-2 (IL-2) together with key mediators of inflammation [98].

Mesalamine interferes with the binding of IFN-γ to its receptor which eventually interrupt the action of IFN-γ and impair the barrier function of intestinal epithelial cells [99]. It also reduces the production of interleukin-2 (IL-2) and subsequently inhibits the proliferation of T-lymphocytes. As this occurs, mesalamine may also cause reduction of IL-1β [100] and TNF-α as both of these cytokines are being produce by IL-2-stimulated peripheral blood mononuclear cells (PBMCs) [101]. These mechanisms of action are related to enhanced activity of hydrogen sulfide (H_2_S) released by derivative of mesalamine [102].

**Figure 3:**
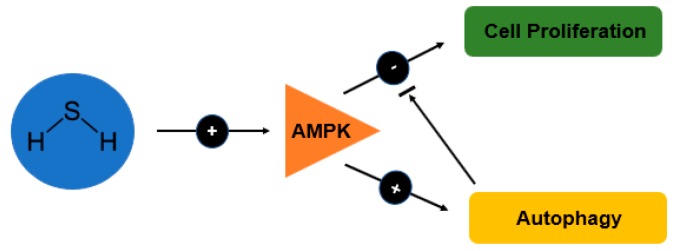
Schematic diagram showing the effects of H_2_S on autophagy and cell proliferation in relation to the AMP-activated protein kinase (AMPK) signalling pathway.

H_2_S is a gaseous bioactive substance that reaches high level in large intestine and usually involved in the regulation of chloride secretion [103], visceral nociceptive processing [104] and mobility in the colon cells (Teague et al., 2002) [105]. As autophagy is being induced by AMPK and inhibited by mTOR, study done by Wu et al. [86] demonstrated that H_2_S increases the phosphorylation of AMPK and this subsequently cause reduction in the phosphorylation of mTOR (Figure 3). The signalling of both AMPK/mTOR cascade eventually render the proliferative effect of H_2_S.

The modulation of inflammation by H_2_S is believed due to its suppression mechanism that reduces the expression of several key proinflammatory cytokines at mRNA level [87]. In inflammatory bowel disease, several non-steroidal anti-inflammatory drugs have been recognized to release H_2_S such as diclofenac, indomethacin, ATB-337, a derivative of diclofenac and ATB-343, a derivative of indomethacin [106]. It was found that H_2_S-releasing derivatives of anti-inflammatory drugs reduce infiltration of leukocytes and their effect was significantly greater than the parent drugs [87,107].

ATB-429, a derivative of mesalamine has potential therapeutic effects as it enhanced an anti-inflammatory activity similar as mesalamine. The analgesic effect of ATB-429 is more superior compare to mesalamine in reducing colorectal distention-induced visceral pain [87]. This becomes an attractive feature of ATB-429 as it has been recently reported to be useful in the treatment of IBD [108]. Fiorucci and colleagues also found that ATB-429 could reduce granulocyte infiltration and proinflammatory cytokines (e.g., TNF*α*, IFN*γ*, IL-1) which indicates a greater efficacy compared to mesalamine.

As discussed earlier, CD management must be by personalised approach. Due to the involvement of many factors, it is necessary for individual genotyping which have CD. Therefore, if there is any defect in the signalling pathway of autophagy, we can target this and modulate the autophagy. Whereas if the defect is more towards the autophagy genes such as ATG16L1, IRGM and NOD2 which cause abolishment of the overall processes, autophagy cannot be stimulated, and this option needs to be excluded from the treatment regime. Another option is by doing gene therapy, which can be done by the replacement of the defect gene. This option however is essentially ineffective and expensive as not all patient with autophagy genes defect is having CD and vice versa. Although it is very difficult to get replacement genes in human body, a recent study by Sander et al. [109] is showing a promising future therapy for IBD.

For the time being, the used of mesalamine, particularly its derivative (ATB-429) with the combination of rapamycin might be useful in the treatment of CD as it gives a promising therapeutic effect by inducing autophagy and could be effective in patients with AIEC as it reduces TNF*α*. This would help in controlling the replication of AIEC moreover in patient with defect of autophagy (e.g., NOD2, IRGM, ATG16L1). A better understanding regarding AIEC with butyrate might support the theory that butyrate could express the virulence genes of AIEC therefore restriction of carbohydrate in the diet of CD patient could be introduce.

A randomized double-blind study found that combination of oral sodium butyrate may improve efficacy of Mesalamine in UC patient [110]. This might be due to the reduction of leukocytes infiltration in colon mucosa by butyrate. Administration of oral butyrate also attenuates the inflammatory profile of intestinal mucosa. Another study showed that oral butyrate is safe and well tolerated for inducing an improvement in CD [111]. Hence, it has high potential to act as an adjuvant to the current treatments.

## 7. Conclusions and Future Perspective

Autophagy is a process of self-digestion which functions to eliminate unwanted cellular material such as damaged organelles or proteins where their accumulation would be toxic to the system. Autophagy also acts as a defense mechanism against invading pathogens and is an integrated part of innate and adaptive immunity. It plays an important role in regulating physiological processes such as development, differentiation and tissue remodeling, which are essential in maintaining cellular homeostasis.

Moreover, studies have shown that defects in autophagy are linked to a myriad of diseases such as heart and liver disease, myopathies, neurodegenerative disease, cancer and CD. Furthermore, defects in autophagy genes constitute one of the strongest risk factors which cause diseases. Autophagy is regulated and activated by nutrient conditions and growth factors.

There is also a strong correlation between the regulation of autophagy and apoptosis as they share interactions between their Bcl-2 family proteins which activate and inhibit both pathways. Inhibition of apoptosis by dissociation of Beclin 1 from Beclin-1 and Bcl-2 complex leads to the activation of autophagy and vice versa. There are dual roles of autophagy which present costs and benefits with respect to cancer, such as the activation of autophagy by Beclin 1 being necessary to inhibit tumour growth. However, this activation may cause treatment resistance and dormancy in tumour cells.

In view of CD, the stimulation of autophagy is necessary as this will lead to reduction of inflammation in CD by inhibiting production of TNF-α caused by AIEC together with its replication. Interestingly, instead of using an autophagy inducer which is still in clinical trial known, such as rapamycin, the current medication being approved by FDA for CD shows a promising autophagy induction. This drug, mesalamine reduce the production of several pro-inflammatory cytokines such as IFN-γ, interleukin 1- IL-1β and IL-2 together with key mediators of inflammation.

Moreover, as it inhibits T lymphocyte proliferation, mesalamine also reduces the level of TNF-α. The reason behind this is due to the production of H2S by Mesalamine was found to induce AMPK and subsequently inhibit mTOR pathway. Furthermore, ATB-429, a derivate of mesalamine is showing a greater efficacy with similar anti-proliferative effect in colon epithelial cells making it a promising potential therapeutic drug to be used in CD. We then suggest that it might be beneficial using mesalamine and rapamycin together with oral butyrate for combination treatment as it has potential synergistic effects.

As autophagy is linked to various diseases, this review points out that there is a need for future study regarding its modulation in these diseases. Currently, there are many studies which emphasize the development of new drugs that modulate this pathway. However, rather than focusing on the new drugs, there are many opportunities to study different drugs available in the market as the mechanism of actions are barely unknown. Moreover, as the drugs are already approved by FDA and have been used for a long time, the safety and efficacy profile is already well established.

The modulation of autophagy is important, but its therapeutic role varies among diseases. In CD, the activation of autophagy is useful to facilitate apoptosis. In addition, the modulation of autophagy shows great promises for future therapeutic approach especially CD. However, as the pathogenesis of CD is complex, personalized treatment becomes a priority as each individual varies and may respond differently according to different approaches.

## Figures and Tables

**Table 1:** Current drugs in CD and their modulation in autophagy. Only the mechanism of action by 5-ASA and anti-TNF- α are known in modulation of autophagy in CD. The newest two classes which are anti-IL-23 and anti-Integrin are correlated with anti-TNF- α.

Drugs	Modulation of Autophagy	Mechanism of Actions	References
**5-ASA** Mesalamine/Sulfasalazine	Induction of autophagy	Lowers proliferation and induces protective autophagy in colon epithelial cells by releasing hydrogen sulphide (H_2_S) via AMPK and inhibition of mTOR and also reduce expression of TN- α	Wu et al. [86] Fiorucci et al. [87]
**Corticosteroids**	Inhibition of autophagy	Suppressed the accumulation of LC3 II in macrophages after mycobacterial infection in Tuberculosis	Wang et al. [88]
**Antibiotics** Metronidazole & Ciprofloxacin	Induction of autophagy	Reduce the expression of *Ang4*, *Retnlb*, *Itln1* and *Itln2* by the gut microflora and bacteria. The deficiency of Atg7 causing less efficient mucous secretion that exacerbated colitis by invasion of microbiota	Tsuboi et al. [89]
**Clarithromycin**	Inhibition of autophagy	Increases TKI-induced cell death in chronic myeloid leukaemia cells. More effective for chemotherapy	Carella et al. [90]
**Thiopurines** Azathioprine, 6- Mercaptopurine, Methotrexate	Induction of autophagy	Immune suppression and protective roles in hepatocytes	Guijarro et al. [88]
**Anti-TNF-α** Infliximab, Adalimumab, Certolizumab pegol	Induction of autophagy	Anti-TNF which acts as immune suppression (targets the inflammatory initiator TNF)	Nys et al. [85]
**Anti-IL-23** Ustekinumab	Induction of autophagy	Inhibit intracellular signaling that promotes autoimmune inflammation such as IL-17, IL-1, IL-6, and TNF-α	Jauregui-Amezaga et al. [91]
**Anti- Integrin** Natalizumab, Vedolizumab	Induction of autophagy	Inhibit the action of integrins thereby decreasing the trafficking of immune cells to the endothelium and suppressing the recruitment of inflammatory cells such as lymphocytes to intestinal lesions	Park and Jeen [92]

ASA: Aminosalicylic acid, AMPK: AMP-activated protein kinase, TKI: Tyrosine kinase inhibitor, TNF: Tumor necrosis factor.
